# Fasudil mediates neuroprotection in ischemia/reperfusion by modulating the ROCK-PPARα-NOX axis

**DOI:** 10.1590/acb387023

**Published:** 2023-12-01

**Authors:** Xitong Yang, Guangming Wang

**Affiliations:** 1 Xitong Yang, Master, Genetic Testing Center, The First Affiliated hospital of Dali University, Dali, China; 2 Guangming Wang, PhD, Professor, Genetic Testing Center, The First Affiliated hospital of Dali University, Dali, China

**Keywords:** Brain Ischemia, fasudil hydrochloride, Peroxisome proliferator-activated receptor alpha, Rho-associated protein kinase

## Abstract

**Purpose:**

Cerebral ischemia-reperfusion (I/R) is a neurovascular disorder that leads to brain injury. In mice, Fasudil improves nerve injury induced by I/R. However, it is unclear if this is mediated by increased peroxisome proliferator-activated receptor-α (PPARα) expression and reduced oxidative damage. This study aimed to investigate the neuroprotective mechanism of action of Fasudil.

**Methods:**

MCAO (Middle cerebral artery occlusion) was performed in male C57BL/6J wild-type and PPARα KO mice between September 2021 to April 2023. Mice were treated with Fasudil and saline; 2,3,5-Triphenyltetrazolium chloride (TTC) staining was performed to analyze cerebral infarction. PPARα and Rho-associated protein kinase (ROCK) expression were detected using Western blot, and the expression of NADPH subunit Nox2 mRNA was detected using real-time polymerase chain reaction. The NADPH oxidase activity level and reactive oxygen species (ROS) content were also investigated.

**Results:**

After cerebral ischemia, the volume of cerebral necrosis was reduced in wild-type mice treated with Fasudil. The expression of PPARα was increased, while ROCK was decreased. Nox2 mRNA expression, NADPH oxidase activity, and ROS content decreased. There were no significant changes in cerebral necrosis volumes, NADPH oxidase activity, and ROS content in the PPARα KO mice treated with Fasudil.

**Conclusions:**

In mice, the neuroprotective effect of Fasudil depends on the expression of PPARα induced by ROCK-PPARα-NOX axis-mediated reduction in ROS and associated oxidative damage.

## Introduction

Stroke is a sudden disorder of cerebral circulation that may either be ischemic or hemorrhagic. It is a notable health problem, with a worldwide prevalence of 80.1 million cases in 2016, and an incidence of 13.7 million cases; ischemic stroke accounted for 84.4%. Although the incidence of stroke has decreased globally, it is still rising in low and middle-income countries, particularly in China^1^.

Although the cause of stroke remains unclear, in most cases genetic predisposition, aging, oxidative stress, and environmental factors are believed to contribute to its occurrence and progression^2^^,^ 3. Reperfusion after ischemia may aggravate damage to the brain tissue, and studies have shown that oxidative stress is one of the key mechanisms causing ischemia-reperfusion (I/R) injury.

The main cause of oxidative stress is the accumulation of reactive oxygen species (ROS), which leads to cell and mitochondrial membrane injury and DNA degradation, ultimately promoting apoptosis^4^. NADPH oxidase is one of the main sources of ROS production in cases of I/R injury^5^. As a key component of the electron transport chain, Nox2 plays an important role in the plasma membrane and is a major inducer of stroke^6^. The major ROS involved in oxidative stress include superoxide anions, hydrogen peroxide, and hydroxyl radicals. Nox2 mostly produces hydrogen peroxide^7^.

As a Rho-associated protein kinase (ROCK) inhibitor, Fasudil delays neuronal death by inhibiting the activity of Rho kinase and prolonging the treatment time window^8^. Statins and Fasudil also mediate transactivation of peroxisome proliferator activated receptor alpha (PPAR-α), which enhances ROS detoxification defenses, protecting cells from cytokine toxicity. PPAR-α agonists that can cross the blood-brain barrier may be administered alone or in combination with Fasudil in neurodegenerative disorders^9^. It is unclear whether Fasudil reduces ROS concentration in ischemia reperfusion models. However, reports suggest that it has neuroprotective effects in cases of I/R injuries^10^; this neuroprotective effect may depend on activation of PPARα^11^^,^ 12.

PPARα belongs to the nuclear receptor superfamily and is a transcription factor activated by ligands. Fasudil promotes the expression of PPARα in oligodendrocytes^9^, thereby increasing the expression of superoxide dismutase (SOD); this may in turn promote the proliferation of vascular endothelial cells and angiogenesis^13^. Cells may produce ROS in response to oxidative stress; excessive ROS exert cytotoxic effects and promote apoptosis, necrosis, and inflammatory responses. In view of their antioxidant activity in eliminating ROS, SODs may reduce apoptosis and inflammatory responses to a certain extent^14^.

Studies have been performed to examine the neuroprotective effects of Fasudil both in vitro and in vivo. However, it is unclear whether these effects are mediated by increased PPARα expression and reduced oxidative damage. Therefore, this study investigated whether the neuroprotective effects of Fasudil was related to the activation of PPARα using wild-type and PPARα knockout mice.

## Methods

### Animals and chemicals

A total of 22 wild-type (WT) specific-pathogen-free male C57BL/6J mice (6–8 weeks old, weighing 20–25 g) were purchased from the Hunan Silaike Jingda Laboratory Animal Co. Ltd. (Hunan Changsha); Certificate number: SCXK-2016 (Xiang)-000. Twelve PPARα knockout male mice were also purchased from the Jackson Laboratory (weighing 20–25 g, aged 6–8 weeks old).

The experiments were approved by the Biomedical Ethics Committee of our university and conducted in accordance with the guidelines for animal experiments issued by the National Institutes of Health. The mice were fed standard food particles and tap water; the indoor temperature was maintained at 20–25°C. The animals were housed in polypropylene cages, maintained in a 12-hour light/dark cycles.

2,3,5-Triphenyltetrazolium chloride (TTC) and Fasudil were purchased from Sigma Aldrich (St Louis, MO, United States of America), the protein extraction kit was purchased from Thermo Scientific (Waltham, MA, United States of America), and the protein concentration assay kit was purchased from Bio-Rad (Richmond, CA, United States of America). The primer synthesis, total RNA extraction, and cDNA synthesis kits were purchased from Invitrogen (Carlsbad, CA, United States of America), and the enzyme activity detection reagents were purchased from Wako (Richmond, CA, United States of America). The real-time fluorescent quantitative polymerase chain reaction (PCR) kit was purchased from Bio-Rad (Richmond, CA, United States of America). All other chemicals were manufactured by Sigma Aldrich (St Louis, MO, United States of America).

### Animal grouping and drug treatment

The WT and PPARαKO mice were randomly divided into two groups, namely, the Fasudil and saline groups; with 10 and six mice, respectively. Fasudil and normal saline (NS) were administered at the doses of 30 mg/kg and 10 mL/kg, respectively, by gavage^15^. Fasudil was dissolved in saline to form a suspension prior to administration. The four groups of mice received the drugs or saline simultaneously, once a day, for three days, and surgery was performed within 1 hour after the last dose.

### Animal analysis

The following parameters were measured: infarct volumes (in 12 WT NS, 10 WT Fasudil, and six PPARαKO mice in each group), PPARα and ROCK activity (in six mice of each WT group), Nox2 mRNA expression levels (in five mice of each WT group), NADPH oxidase activity measurement (in five mice of each WT and PPARαKO group), and ROS content (in five mice of each WT and PPARαKO group).

### Middle cerebral artery occlusion

The mice were anesthetized using 1.5% isoflurane during surgery, and their rectal temperature was maintained at 37 ± 1°C using a heating pad. The right common and external carotid arteries were separated under direct visualization using a surgical microscope, and ligated. A 8-0 nylon thread monofilament measuring 11 mm in length, coated with silicone resin and hardener mixture (Heraeus, Hanau, Germany), was inserted into the internal carotid artery via the common carotid to block the blood flow of the middle cerebral artery; the timing began when the cerebral blood flow (CBF) dropped below 20% of the original value.

After 60 minutes, the nylon thread was removed to allow reperfusion. During the operation, the CBF was monitored using a detector (BD diagnostics), which was placed on the surface of the cranium over the ischemic area, behind the margin of the left ear. The animals were humanely sacrificed 18 hours after reperfusion, and the brain tissue was obtained for follow-up tests.

### Infarct volume measurement

The brain was collected by separating it from the bone and meningeal membranes of the severed head; five coronal sections of 2-mm thickness were prepared from the brain tissue and stained with 2% TTC for 30 minutes at room temperature. The infarct area was analyzed using the MCID image analysis system (Imaging Research, Inc., St Catherines, Ontario, Canada).

### Western blotting

Total protein was extracted from the brain tissue using a protein extraction kit, and the concentrations were measured using the Bio-Rad protein assay kit; sodium dodecy l sulfate-polyacrylamide gel electrophoresis was performed using 15 μg/5 µL of the protein (separation gel concentration: 8%), and transferred to a polyvinylidene difluoride (PVDF) membrane by the Bio-Rad semi-dry transfer system, after washing it with 0.1 M tris-buffered saline (TBS). This was then incubated at room temperature for 30 minutes after adding 1% skimmed milk powder.

Rabbit anti-mouse PPARα, ROCK polyclonal antibody (Cayman, Ann Arbor, MI, United States of America) (1:5,000), and goat anti-mouse β-actin monoclonal antibody (Sigma-Aldrich, St. Louis, MO, United States of America) (1:2,000) were added and incubated with the membrane overnight at 4°C. After washing thrice with 0.1 M TBS, the membrane was incubated with anti-goat or rabbit secondary antibody (IRDye 800CW and IRDye 680CW label, respectively; 1:3,500).

After washing and scanning with the Bio-Rad Western blot detection system, the images were analyzed using Image J (Image J 1.46r, Wayne Rasband, National Institutes of Health, United States of America). The ratio of PPARα and ROCK to β-actin were evaluated; the gray values represented the relative values of PPARα and ROCK expression.

### Measurement of Nox2 mRNA expression

Real-time PCR was used to analyze the Nox2 mRNA levels. Total RNA was extracted using TRIzol reagent (Invitrogen); cDNA was then synthesized using a reverse transcription kit (Invitrogen), and PCR was performed using the iQ SYBR Green kit.

The cycle conditions were as follows: initial denaturation at 95°C for 10 minutes, followed by denaturation at 95°C for 10 seconds, annealing at 55°C for 10 seconds, with extension for 15 seconds at 72°C. The cycle was repeated 30 times.

The primers sequences included a forward primer: 5’-ACTGCGGAGAGTTTGGAAGA-3’ and reverse primer: 5’-GGTGATGACCACCTTTT GCT-3’; the amplified fragment length was 201 bp. The data were analyzed using the 2^ΔΔCT^ method. The value for saline control group was considered as 1; this was used as the reference for comparison with the Fasudil group. The magnitude of changes reflected the Nox2 mRNA levels.

### Measurement of NADPH oxidase activity

Lucigenin (10 µMol/L) and NADPH (100 µMol/L) were added to the ischemic brain tissue homogenate supernatant, and chemiluminescence microarray (Luminometer) was used for determination, continuously for 5 minutes, once every 30 seconds. The value in the saline group was defined at 100 for comparison with the Fasudil group.

### Measurement of reactive oxygen species levels

A tissue suspension was prepared using the brain tissue and Locke buffer (40 mg/L) as per the kit instructions. The suspension was incubated at 37°C for 30 minutes after adding the dichloro-dihydro-fluorescein diacetate (DCFH-DA) probe (1 µL/L). Non-luminous DCFH-DA can penetrate the cell membrane freely, and intracellular ROS can oxidize DCFH-DA to fluorescent DCFH; therefore, the intensity of the fluorescence is proportional to the magnitude of intracellular ROS activity. The intensity was detected using a fluorescent microplate at excitation and emission wavelengths of 480 and 530 nm, respectively.

### Statistical analysis

All experimental data are presented as means ± standard deviation (SD). The GraphPad Prism 5.00 for Windows (GraphPad Software, La Jolla, CA, United States of America) was used to analyze the data. The Statistical Package for the Social Sciences 17.0 software was used to perform one-way analysis of variance (ANOVA) prior to the t-test. P < 0.05 was considered statistically significant.

## Results

### Fasudil reduced the infarct volume in wild-type mice

**Figure 1:**
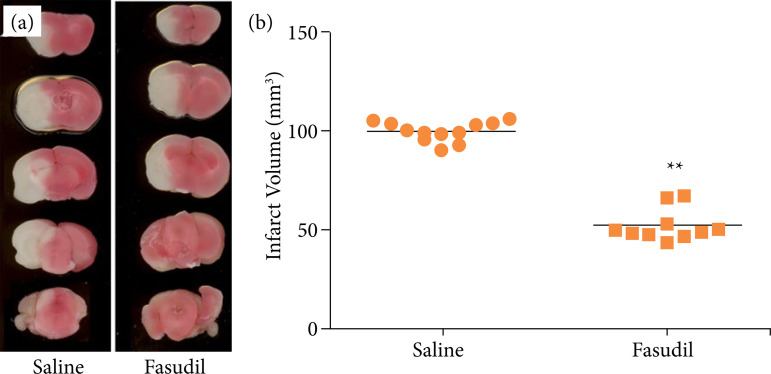
Neuroprotective effect of Fasudil after ischemia/reperfusion. **(a)** Representative case from each treatment group showing five serial coronal brain slices following 2% 2,3,5-Triphenyltetrazolium chloride in wild-type mice; **(b)** necrosis volumes of brain tissue.

White infarcts were observed on TTC staining of the I/R brain tissue in the NS control group of WT mice; a marked decreased was observed after treatment with fasudil (Fig. 1a); image analysis demonstrated cerebral infarction volumes of 99.926 ± 4.819 and 52.190 ± 7.570 mm^3^, respectively (Fig. 1b). The t test confirmed significant differences in volume reduction (P < 0.01). However, there were no changes in body temperature and CBF between the two groups, before and after perfusion (Table 1). The results indicate that Fasudil may reduce infarct volumes after I/R and improve symptoms of brain injury.

**Difference compared with saline group, P<0.01.:

**Table 1:** Regional cerebral blood flow and body temperature.

		Saline (n = 12)	Fasudil (n = 10)
% Regional cerebral blood flow	Before	100	100
	After	10.80 ± 4.54	10.08 ± 2.20
	Reperfusion	95.52 ± 2.37	98.06 ± 2.56
Body temperature	Before	36.50 ± 0.43	36.39 ± 0.10
	After	36.17 ± 0.08	36.43 ± 0.51
	Reperfusion	36.34 ± 0.13	36.26 ± 0.14

Fasudil enhanced PPARα expression and decreased ROCK expression

**Figure 2:**
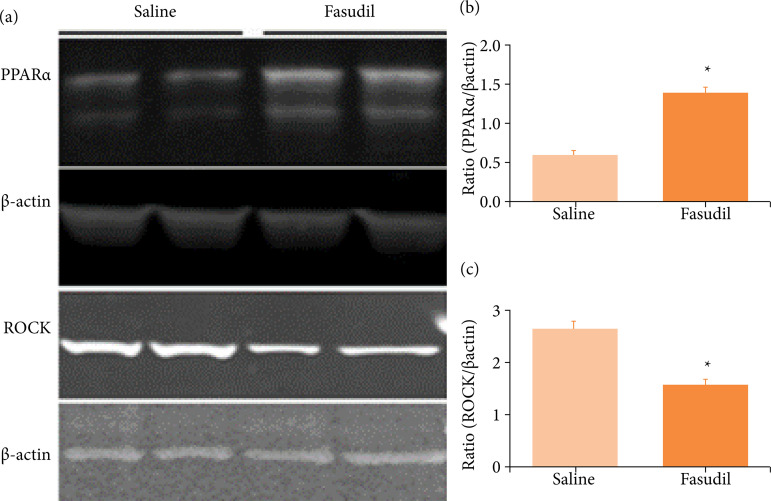
Effects of Fasudil on PPARα and ROCK expression in wild-type mice. **(a)** Western blotting shows the expression of PPARα and ROCK after Fasudil treatment; **(b)** Image J analysis shows the expression of PPARα and ROCK after Fasudil treatment.

We measured the PPARα and ROCK levels in the ischemic tissue to evaluate the impact of Fasudil on PPARα and ROCK expression. As shown in Fig. 2, the PPARα expression level in the saline group was 0.614 ± 0.042; the value was significantly increased to 1.417 ± 0.050 in the Fasudil group. The level of ROCK expression in the saline group was 2.569 ± 0.111; this was significantly reduced to 1.505 ± 0.122 in the Fasudil group. These results indicate that Fasudil promoted the expression of PPARα and reduced ROCK expression after I/R.

PPARα: peroxisome proliferator-activated receptor-α; ROCK: Rho-associated protein kinase; *difference compared with the saline group P < 0.01.

### Fasudil inhibits the expression of Nox2 mRNA, decreases NADPH oxidase activity, and ROS content

**Figure 3:**
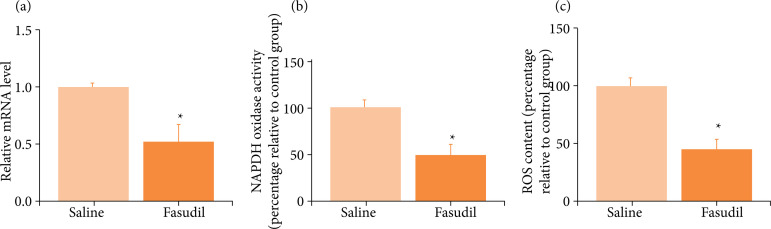
Effects of Fasudil treatment on Nox2 mRNA, NADPH oxidase activity, and ROS content in wild-type mice. **(a)** Nox2 mRNA level; **(b)** NADPH oxidase activity; **(c)** ROS content.

In the I/R tissue treated with Fasudil, the Nox2 mRNA expression level was 0.524 ± 0.142, which was significantly lower than that of the saline group at 1.000 ± 0.045. The NADPH oxidase enzymatic activity level at 49.390 ± 10.304 and ROS content at 46.590 ± 6.912 were markedly below those of the saline group at 100.000 ± 7.091 and 100.000 ± 6.606, respectively. These findings suggest that, after I/R, Fasudil can reduce the level of Nox2 mRNA and decrease NADPH oxidase activity and ROS content. The results are shown in Fig 3.

ROS: reactive oxygen species; *difference compared with the saline group P < 0.01.

### Fasudil has no protective effects on ischemia-reperfusion injuries in PPARαKO mice

**Figure 4:**
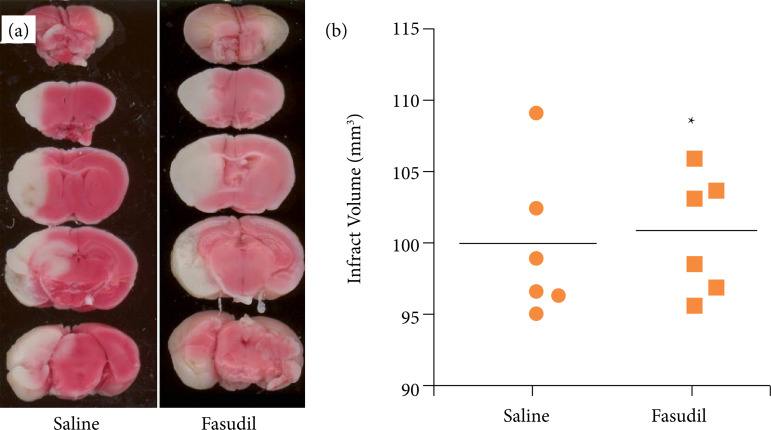
Fasudil has no neuroprotective effect after ischemia-reperfusion. **(a)** Five serial coronal slices of ischemia-reperfusion brain tissue in PPARαKO mice (one mouse per group) stained with 2% 2,3,5-triphenyltetrazolium chloride; **(b)** infarct volumes of brain tissue necrosis.

On TTC staining of the tissue slices after I/R, the brain tissue of the saline group showed white infarcts (99.901 ± 4.792 mm^3^); no changes were observed compared with the saline group of WT mice (99.926 ± 4.819 mm^3^) (Figs. 1 and 4). After treatment with Fasudil, the white infarct volumes were not altered in the PPARαKO mice (100.823 ± 3.798 mm^3^) (Fig. 4), and the t test did not indicate any significant differences between the groups. Since the infarct volumes and degrees of injury remained unaltered in the PPARα KO mice treated with Fasudil, we concluded that it exerted no protective effects in PPARα KO mice.

PPARα: peroxisome proliferator-activated receptor-α; *compared with saline group, P > 0.05.

### Fasudil did not affect the NADPH oxidase activity and reactive oxygen species content in PPARαKO mice

**Figure 5:**
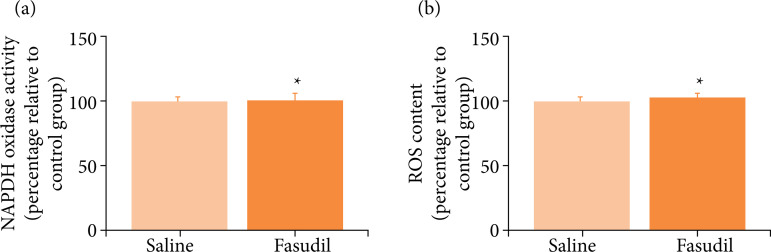
Effect of Fasudil treatment on NADPH oxidase activity and ROS content in PPARαKO mice. **(a)** NADPH oxidase activity; **(b)** ROS content.

After I/R, the NADPH oxidase activity and ROS content in the PPARαKO mice from the saline group were 98.000 ± 4.022 and 99.200 ± 4.240, respectively; no changes were observed compared with the saline group of WT mice. After treatment with Fasudil, the activity level of NADPH oxidase and the content of ROS were found to be 101.390 ± 4.077 and 102.590 ± 2.950 (Fig. 5), respectively; no differences were noted between the two groups.

ROS: reactive oxygen species; PPARα: peroxisome proliferator-activated receptor-α; *compared with saline group, P > 0.05.

## Discussion

The findings in this cohort suggest that the neuroprotective effect of Fasudil in brain tissue is mediated by the regulation of PPARα gene expression, which reduces oxidative stress.

Cerebral I/R refers to a series of reactions that occur after blood flow is resumed following cerebral ischemia; these reactions aggravate brain tissue damage or render it irreversible. Local cerebral ischemia produces a strong inflammatory response with the release of a large number of inflammatory cells, that increase the production of oxygen free radicals and other inflammation-related products, with consequent brain tissue injury, vascular endothelial dysfunction, and neuronal cell apoptosis. Therefore, the regulation of inflammatory cell activation and the release of related factors is the key to alleviating ischemic brain injury^16^.

Fasudil, as a ROCK inhibitor, plays a key role in inhibiting secondary injury after ischemia. It also inhibits the production of neutrophils and oxygen free radicals in blood vessels, and the development of an inflammatory response^17^. This study, based on the middle cerebral artery occlusion model, confirmed that the neuroprotective effects of Fasudil after I/R is associated with the activation of PPARα; Fasudil had no neuroprotective effects following brain injury in PPARα knockout animals.

PPARα regulates the metabolism of adipose tissue and amino acids and is implicated in abnormal lipid metabolism regulation, atherosclerosis, coronary heart disease, and other pathologies^18^. In view of PPARα pivotal role, the activity of NADPH oxidase and the content of ROS were measured in this study to determine whether Fasudil depends on PPARα activation to inhibit NADPH oxidase activity, alter Nox2 mRNA levels, and reduce ROS content.

The results confirmed that the infarction volumes in WT mice after I/R were significantly lower compared to the Fasudil group. The expression of PPARα significantly increased, and the expression of ROCK was reduced markedly, while the levels of Nox2 mRNA, NADPH oxidase, and ROS content were conspicuously lower than those of the saline group. Fasudil reduced oxidative damage and brain injury, demonstrating a neuroprotective role.

A research found that, in neurons lacking or with inhibited function of NADPH oxidase, peroxide production is minimal; both NADPH oxidase activity and protein expression levels reflect the ROS producing ability of brain cells^19^. The NOX2 subtype of NADPH oxidase is the primary producer of intracerebral ROS. Inhibition of ROS production may reduce infarct volumes, mitigate blood brain barrier damage, and improve brain function^20^^,^ 21. The mRNA and protein expressions of Nox2 and relevant regulatory subunits are increased after stroke. ROS produced owing to increased NOX2 enzyme activity may lead to vascular inflammation, leukocyte accumulation, oxidative damage, and cell death, thereby enlarging the ischemic infarct^22^. Research reports suggest that PPARα is widely expressed in brain tissue; in mice, Nox expression and ROS content may be inhibited in cases of I/R injuries of the brain, to protect against related damage^23^. Therefore, restricting NADPH oxidase activity may effectively reduce ROS content in vivo, reducing I/R injury-induced damage.

SOD is an important antioxidant enzyme in vivo that removes superoxide anion free radicals, reduces lipid peroxidation, and prevents cell and tissue damage; as a protective cytokine, it plays an important role in the oxidation and anti-oxidation balance^24^. Previous studies have found that PPARα positively regulates the content and activity of SOD, thereby playing a protective role in I/R injury^25^. In this study, the PPARα expression in WT mice increased after Fasudil treatment. The associated decrease in NADPH oxidase and ROS content owing to an increased expression of PPARα alleviated the I/R brain injury, thereby contributing to the neuroprotective effect of Fasudil.

Previous studies have shown that, in cerebral I/R injury models, the infarction volumes and neurological deficits are more severe in PPARα knockout mice than those of the WT animals^26^. In this study, the infarct volumes, NADPH oxidase activity, and ROS content in the PPARα knockout mice of the saline control and Fasudil groups were 99.901 ± 4.792 and 100.823 ± 3.798 mm^3^, 98.000 ± 4.022 mm^3^ and 101.390 ± 4.077 mm^3^, and 99.200 ± 4.240 and 102.590 ± 2.950 mm^3^, respectively; this concurred with the findings of previous studies. In summary, the infarction volume, enzyme activity, and ROS content showed no significant changes, and the oxidative stress and brain damage were unchanged. We concluded that Fasudil has no neuroprotective effect in PPARαKO mice.

Therefore, the lack of Fasudil-induced neuroprotection in PPARαKO mice after cerebral ischemia may be attributed to PPARα loss-of-function, decreased SOD expression, and increased ROS concentration. Oxidative damage is not prevented, thereby offering no neuroprotection against brain injury.

## Conclusion

Our findings in this case-control study in mice demonstrated that the neuroprotective effect of Fasudil after I/R is dependent on PPARα. The increased levels of PPARα inhibited NADPH oxidase activity, reduced the production of ROS, and reduced oxidative damage. The role of the ROCK-PPARα-NOX axis in neuroprotection is therefore demonstrated. Further studies in larger cohorts and other animal models are needed to validate our findings.

## Data Availability

All dataset were generated or analyzed in the current study.
